# Animal models of sarcopenia

**DOI:** 10.1111/acel.13223

**Published:** 2020-08-28

**Authors:** Courtney J. Christian, Guy M. Benian

**Affiliations:** ^1^ Department of Pathology Emory University Atlanta Georgia USA

**Keywords:** aging, *Caenorhabditis elegans*, *Drosophila*, mice, muscle, rats, sarcopenia, zebrafish

## Abstract

Sarcopenia is the age‐related decline in muscle mass and function without any underlying disease. The exact molecular mechanisms responsible for this pathology remain unknown. The use of model organisms, such as mice, rats, flies, and worms, has advanced the field of sarcopenia research by identifying therapeutic strategies and genetic mutations that result in improved muscle mass and function of elderly animals. This review discusses molecular and therapeutic discoveries made using these model organisms and how these animals can be further utilized to better understand sarcopenia pathogenesis. In rodents, flies, and worms, dietary restriction improves muscle performance in old animals. In rodents and worms, exercise and a number of naturally occurring compounds alleviate sarcopenia. Reduction in the insulin/IGF1 receptor pathway, well known to promote longevity, improves sarcopenia in worms and flies. Mitochondrial dysfunction may contribute to the pathogenesis of sarcopenia: In rodents, there is age‐dependent reduction in mitochondrial mass and a change in morphology; in nematodes, there is age‐dependent fragmentation of mitochondria that precedes sarcomeric disorganization. In Drosophila and rats, components of the 26S proteasome are elevated in aged muscle. An advantage of the worm and fly models is that these organisms lack muscle stem cells, and thus processes that promote the maintenance of already assembled muscle, can be identified without the confounding influence of muscle regeneration. Zebrafish are an up and coming model of sarcopenia for future consideration. A better understanding of the molecular changes behind sarcopenia will help researchers develop better therapies to improve the muscle health of elderly individuals.

## Graphical abstract

Sarcopenia is the age‐associated decline in skeletal muscle mass and function without any underlying disease, and is a major contributor to the frailty, immobility, and mortality of the elderly. In this review we report the contributions that model organisms—rodents, *Caenorhabditis* *elegans*, Drosophila, zebrafish—are making towards the understanding of its molecular mechanisms and discovery of potential therapies.

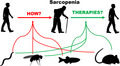

## 1 INTRODUCTION

Sarcopenia, the decline in skeletal muscle mass and function without any underlying disease, is a major contributor to physical disability, poor quality of life, and death among the elderly (Cruz‐Jentoft et al., 2014). 40%–50% of individuals over 80 years of age suffer from this loss of muscle mass and function (Barbosa‐Silva, Bielemann, Gonzalez, & Menezes, 2016; Iannuzzi‐Sucich, Prestwood, & Kenny, 2002). The molecular mechanisms responsible for this age‐related condition remain unknown (Zembron‐Lacny, Dziubek, Rogowski, Skorupka, & Dabrowska, 2014). Resistance training and dietary changes are recognized as the gold standard therapy but have only a modest effect (Candow, 2011). There is a direct association between poor handgrip strength, reduced physical function, and a higher risk of falling (Szulc, Feyt, & Chapurlat, 2016). Intriguingly, even in middle age (40–69), there is a correlation between reduced grip strength and all‐cause mortality and incidence of and mortality from cardiovascular disease, respiratory disease, and cancer (Celis‐Morales et al., 2018). A GWAS of over 200,000 individuals identified 64 genes associated with grip strength, and many of these genes are known to have roles in neural development or brain function (Tikkanen, Gustafsson, & Ingelsson, 2018). Interestingly, one hypothesis about sarcopenia is that it is caused by motor neuron decline with age (Kwan, 2013). Elderly individuals at a higher risk of falling are thus at a higher risk of vertebral and nonvertebral fractures (Szulc et al., 2016)—leading to surgeries, hospitalization, and increased medical complications and risks. Additionally, the increased risk of respiratory illness in individuals over 65 years of age may be partially explained by the aging‐related weakening of the diaphragm muscle resulting in nonproductive coughs and more severe respiratory illnesses (Gosselin, Johnson, & Sieck, 1994). With the ever‐increasing population of elderly and the predicted strain on the healthcare system (Statistics, 2012), it is crucial we understand the molecular mechanisms responsible for age‐related diseases such as sarcopenia so that we can develop more effective therapies and prevention methods.

In order to better tease out the molecular “players” responsible for muscle maintenance and determine how they change with age, we need to look more toward model organisms for insight. Model organisms provide the benefit of shorter lifespans, larger sample sizes, genetic manipulation, and controlled environmental conditions. Currently, the sarcopenia research field has utilized rodents (rats and mice), *Drosophila*, and *C*.* elegans* as models for aging muscle. This review will outline the insights gained using these model organisms as well as discuss the future of sarcopenia research using model organisms.

**Figure 1:**
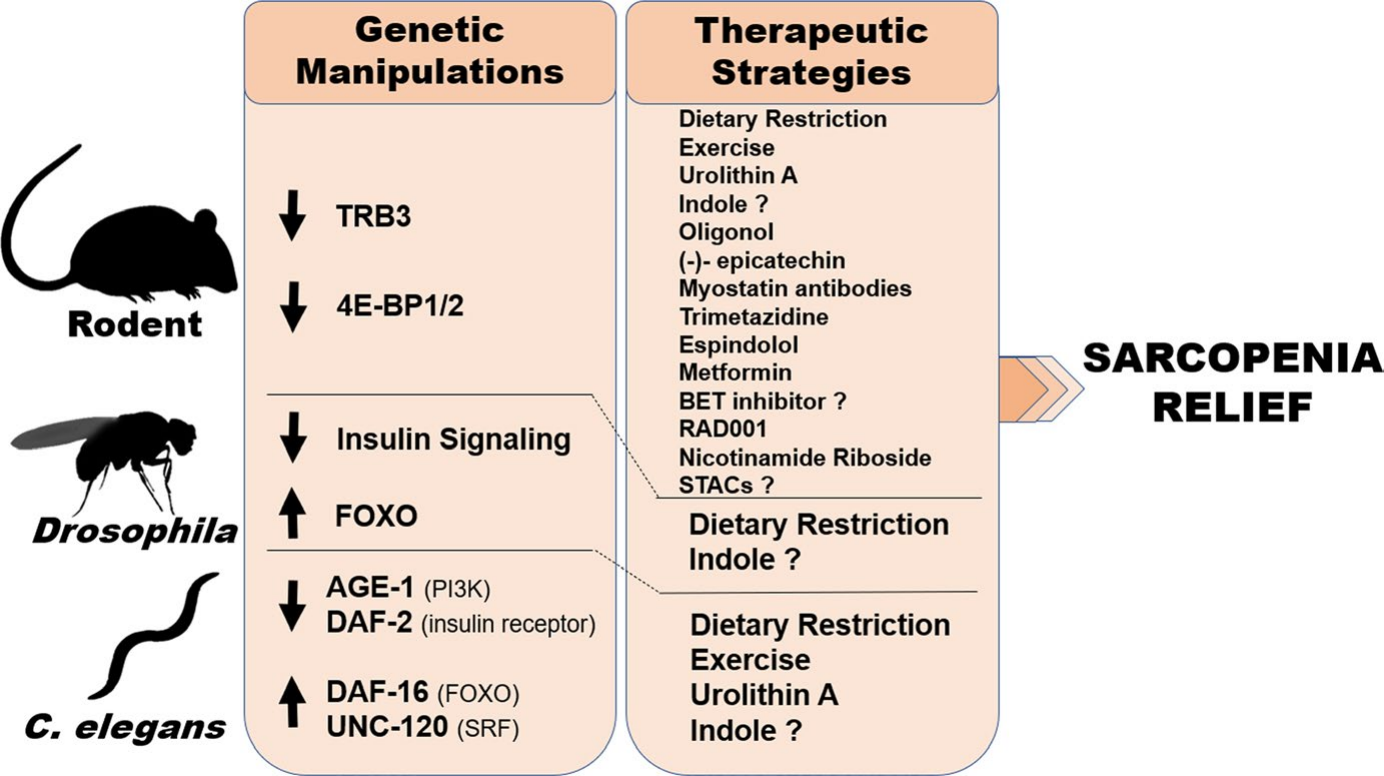
Summary of genetic manipulations and therapeutic strategies that have been found to alleviate sarcopenia in rodents, *Drosophila*, and/or *Caenorhabditis elegans*

## 2 RODENTS

### 2.1 The models

Rats and mice have been the preferred mammals for modeling most human diseases. To standardize rat models used in aging‐related research, the USA National Institute on Ageing (NIA) currently recommends the use of F344 and Brown Norway (BN) inbred rat strains and provides these animals freely for relevant aging‐related research. F344 are considered aged (50% survival) at 24 months, and BN are considered aged at 32 months. The average lifespan of F344 rats is about 31 months in males and 29 months in females (Sass et al., 1975), and the average lifespan of BN rats is 2 years (Lipman, Chrisp, Hazzard, & Bronson, 1996). Other rat strains used include Sprague Dawley (average lifespan: 29–30 months) and Wistar rats (average lifespan: 24 months). The common laboratory mouse *M*.* musculus* has an average lifespan of ~2.5 years (Yuan, Peters, & Paigen, 2011). These mammalian models have the benefit of allowing researchers to study the effects of co‐morbidity—that is, sarcopenia progression in the presence of heart disease, obesity, diabetes, etc. Like humans, mice and rats also have muscle stem cells (satellite cells) and are thus well‐suited for studying the possible contribution of an age‐related decline in muscle regeneration to sarcopenia. The rodent model is also better suited for testing drug and therapeutic efficacy. Some drawbacks of this model include the expensive, time‐consuming, and labor‐intensive efforts needed to obtain large sample sizes. Compared to other models, the rodent model is not easily genetically manipulated—screening for mutants, or generating CRISPR mutants, is either not feasible or time‐consuming and expensive. Finally, it should be mentioned that mammalian skeletal muscle contains two “fiber types” that differ in their rates of contraction and metabolic activities, the type I or slow‐twitch muscle and type II or fast‐twitch muscle. During sarcopenia in humans, there is loss of both type I and type II cells, but of the cells that remain, type II cells undergo more atrophy (Deschenes, 2004). This fiber‐type heterogeneity makes studies of sarcopenia in rodents more difficult to interpret.

### 2.2 Molecular discoveries

In 2010, Altun et al. reported the activity of the ubiquitin–proteasome pathway in aged muscles of Sprague Dawley rats. They found two‐ to threefold higher levels of 26S proteasomes than those of young adult controls. 26S proteasomes purified from muscles of aged and young adult rats showed a similar capacity to degrade peptides, proteins, and an ubiquitylated substrate, but differed in levels of proteasome‐associated proteins (26S proteasome subunits, ubiquitin ligases, and deubiquitylating enzymes were all increased in aged rats). The aged muscles contained higher levels of the ubiquitin ligase CHIP, involved in eliminating misfolded proteins, and MuRF1, which ubiquitylates myofibrillar proteins. Nevertheless, their content of polyubiquitylated proteins was higher than in young adult animals—suggesting that the increase in proteasome‐associated proteins could be due to impaired degradation of these proteins, causing an increase in nonfunctional polyubiquitylated proteins that are neither properly degraded nor functional (Altun et al., 2010). This group also tested the effects of dietary restriction (70% of what the control group was fed—not enough to cause starvation) on ubiquitination and the proteasome pathway in aged rat muscle. They found that reducing the caloric intake of these rats resulted in decreased or complete prevention of the age‐associated increases in ubiquitin–proteasome system components. The dietary restriction rats experienced significantly less muscle atrophy compared with the control rats at 30 months; however, the authors did not comment on muscle function or show any results from motility tests (Altun et al., 2010).

Tribbles homolog 3 (TRB3) is a pseudokinase that acts as a modulator of substrate ubiquitination and as a molecular scaffold for the assembly and regulation of signaling modules (Eyers, Keeshan, & Kannan, 2017). TRB3 was previously reported to exhibit an age‐related increase in expression (Meyer, Schenk, & Lieber, 2013) and play a vital role in cell proliferation, differentiation, and fibrosis. It has been demonstrated that TRB3 caused muscle fiber atrophy and a decrease in muscle function by negatively modulating protein turnover in the condition of food deprivation (Choi et al., 2017, 2019) and could inhibit the myogenic differentiation of C2C12 (mouse myoblast) cells (Kato & Du, 2007). Shang et al. acquired TRB3 knockout mice and found that sarcopenia was attenuated in these mice compared with aged controls via the alleviation of atrophy and fibrosis of skeletal muscles (Shang et al., 2020). The TRB3 knockout mice had reduced atrophy and a greater exercise capacity compared with the wild‐type mice at the same age. The authors did not reveal whether the TRB3 knockout mice lived longer or at what age they may have developed sarcopenia.

As noted below, in *C*.* elegans (*Depuydt et al., 2013; Duhon & Johnson, 1995; Herndon et al., 2002 *)* and in Drosophila (Demontis & Perrimon, 2010; Owusu‐Ansah, Song, & Perrimon, 2013), genetic reduction in the insulin/IGF1 signaling pathway, which causes increased nuclear translocation of DAF‐16 (FOXO in mammals), results in lifespan extension and reduced sarcopenia in these organisms. Although similar experiments have not yet been conducted on rodent muscle, a mouse in which FOXO 1, 3, and 4 genes were knocked out in neurons resulted in age‐associated axonal tract degeneration and motor dysfunction (Hwang et al., 2018). The authors also found that the neuron‐specific FOXO KO mice had elevated mTORC1 (mechanistic target of rapamycin complex 1) activity and that mTORC1 inhibition by rapamycin treatment prevented axonal degeneration. Although not reported in this study, it would be interesting to examine the possibility of accelerated sarcopenia in these mice given that one proposed mechanism for sarcopenia is age‐dependent degeneration of motor neurons (Kwan, 2013).

Le Bacquer et al. found that mice deficient in the eukaryotic translation initiation factor 4E‐binding proteins (4E‐BP) 1 and 2 (double knockout) exhibited an increase in muscle mass and grip strength compared with age‐matched (24‐month‐old) controls (Le Bacquer et al., 2019). 4E‐BP1/2 sequesters eukaryotic translation initiation factor 4E unless mTORC1 leads to 4E‐BP phosphorylation and, ultimately, increased cap‐dependent translation initiation. The authors speculate that 4E‐BPs can be utilized as drug targets to increase muscle protein synthesis and protect against age‐associated loss of muscle mass and function. In contrast to the findings discussed above, the data of Le Bacquer et al. may suggest increased mTORC1 activity leads to increased muscle protein synthesis and retention of muscle mass with age. However, as discussed below, Joseph et al have found that in rat sarcopenic muscle, there is increased activation of mTORC1.

Muscle mitochondrial dysfunction likely plays an important role in the pathogenesis of sarcopenia. The age‐dependent reduction in mitochondrial mass is likely to be due in part to a decline in the major regulator of mitochondrial biogenesis, “peroxisome proliferator‐activated receptor‐γ coactivator‐1α” (PGC‐1α), as has been shown for rat muscle (Chabi et al., 2008; Kang, Chung, Diffee, & Ji, 2013). In mice, a carefully performed EM study shows that in sarcopenic muscle the mitochondria have a different morphology from those in younger muscle; depending on location, these mitochondria are either larger and less circular or longer and more branched, suggesting increased fusion and /or decreased fission (Leduc‐Gaudet et al., 2015). Hiona et al. found that mice with increased mitochondrial DNA mutations due to a proofreading‐deficient mitochondrial DNA polymerase experienced greater loss of muscle mass compared with wild‐type littermates (Hiona et al., 2010). The authors suggest that the accumulation of mtDNA mutations with age is a large contributor to sarcopenia pathogenesis. This study suffers from the fact that these mice have increased mtDNA mutations in all their tissues, not just skeletal muscle.

Another set of genes that may prove pivotal to sarcopenia and other age‐related pathologies are the circadian genes. Kondratov et al. have shown that mice lacking the circadian transcription factor BMAL1 (brain and muscle ARNT‐link protein) have reduced lifespans and an early onset of sarcopenia, as well as other signs of premature aging (Kondratov, Kondratova, Gorbacheva, Vykhovanets, & Antoch, 2006). These results are not surprising as BMAL1 regulates several genes involved in tissue homeostasis.

### 2.3 Therapeutic discoveries

The major contribution rodents have made to sarcopenia research has been discovering therapeutics, drugs, and lifestyle changes that can attenuate sarcopenia. Many of the compounds investigated for sarcopenia treatment are naturally occurring. Ryu et al. found that urolithin A, a natural dietary compound found in some nuts and fruit, was able to improve muscle function in aged mice via inducing mitophagy (mitochondrial autophagy) (Ryu et al., 2016). Another group of naturally occurring compounds that may extend the muscle health of mice are indoles, which come from commensal microbiota (Sonowal et al., 2017). Mice treated with indoles retain greater mobility as they age as compared to the age‐matched controls, but they do not experience an extension of lifespan (Sonowal et al., 2017). A limitation of this study is that the authors did not investigate whether muscle mass or structure was preserved on indoles. Chang et al. found that oligonol, an extract from lychees consisting of catechins, procyanidins, and other phenolic compounds, increased skeletal muscle mass and grip strength in 32‐week‐old SAMP8 (senescence‐accelerated mouse prone 8) mice (Chang et al., 2019). The authors postulate that oligonol is a promising compound to treat sarcopenia via improving muscle mitochondria quality. Gutierrez‐Salmean et al. found that the flavanol (‐)‐epicatechin (a compound found in cocoa) treatment decreased myostatin and senescence‐associated β‐galactosidase and increased levels of markers of muscle growth, such as follistatin (Gutierrez‐Salmean et al., 2014).

Myostatin, a TGF family member, is a myokine secreted by skeletal muscle cells that acts to limit muscle cell growth. It does so by binding to an activin type II receptor and inhibiting the differentiation of myoblasts during development, or satellite cells in mature muscle, into differentiated muscle cells (Lee, 2004). While crucial for proper development, myostatin might be detrimental for aging muscles. Multiple groups have found that inhibiting myostatin via antibodies in aged mice improves muscle mass and strength, as well as insulin sensitivity (Camporez et al., 2016; LeBrasseur et al., 2009; Murphy et al., 2011). Ferraro et al. found that trimetazidine (a metabolic modulator that improves the efficiency of glycolysis) administration to aging mice increased muscle strength, expression of slow myosin heavy chain isoform in gastrocnemius muscle, and the number of small‐sized myofibers in tibialis anterior muscle (Ferraro et al., 2016). Potsch et al. (2014) found that the small molecule espindolol significantly increased lean body mass with reduced fat mass in aging rats. Espindolol treatment leads to a reduction in catabolic/atrophic signaling by blocking the chronic activation of the β‐1 adrenergic receptor, while inducing anabolic signaling by the intrinsic sympathomimetic activity effect on the β‐2 adrenergic receptor (Potsch et al., 2014). Another group found that long‐term moderate exercise combined with metformin treatment induced a “hormetic response” (explained below) in Wistar rats that prevented age‐associated loss of muscle strength and muscle mass (Hernandez‐Alvarez et al., 2019). Metformin is known to inhibit hepatic gluconeogenesis by decreasing glucose plasma levels and is often prescribed to treat type II diabetes (Hundal et al., 2000; Wessels, Ciapaite, van den Broek, Nicolay, & Prompers, 2014). It has also been found that metformin inhibits mitochondrial respiratory chain complex I and generates low levels of oxidants, which induce the antioxidant response producing a hormetic effect—a favorable biological response to a light stressor. At the same time, reduced ATP levels activate AMPK (AMP‐activated protein kinase), which activates diverse protective pathways. The pharmacological inhibition of BRD4 (bromodomain protein) using a BET (bromodomain and extraterminal domain) protein inhibitor protected tumor‐bearing mice from muscle wasting by preventing the activation of catabolic genes associated with muscle atrophy (Segatto et al., 2017). While this is a model of cancer‐induced cachexia and not sarcopenia, this does suggest an epigenetic role during muscle wasting and provide credence for investigating epigenetic targets when developing sarcopenia therapeutics.

Consistent with the observed elevation of mTORC1 activity in FOXO KO neurons (Hwang et al., 2018), Joseph et al. reported that in rats there was increased activation of mTORC1 in sarcopenic muscle as measured by increased levels of its effector, phosphorylated ribosomal protein S6 (S6K1) (Joseph et al., 2019). Administering a clinically relevant “low dose” of a compound known to inhibit mTORC1 activity called RAD001, Joseph et al. showed that in some muscles that would have undergone sarcopenia, the mass of that muscle was increased, there was histological evidence of improved muscle morphology (e.g., increases in fiber cross‐sectional area, decreased frequency of centrally placed nuclei), reduced expression of putative atrophy genes (e.g., MuRF1 and MT1), and increases in levels of protein markers of autophagy (which decline with sarcopenia) (Joseph et al., 2019).

SIRT1 is a NAD+‐dependent deacetylase that removes acetyl groups from many different proteins and participates in a wide variety of cellular processes including cell survival, metabolism, DNA repair, and mitochondrial homeostasis. Loss of function of SIRT1 gene orthologs results in reduced lifespan of yeast, *C*.* elegans*, and mice. Studies in *C*.* elegans* and mice show an age‐dependent decrease in both NAD+levels and SIRT1 activity (Mouchiroud et al., 2013). NAD+precursors in the human diet include nicotinamide riboside (NR) and nicotinamide mononucleotide (NMN). Zhang et al. reported that 24‐month‐old C57BL/6J fed a diet supplemented with NR for 6 weeks showed improved muscle function including maximal running times and distances, and limb grip strength. In addition, NR supplementation led to improvements in muscle stem cells including increased numbers of these cells, less DNA damage, and increases in the amounts of OXPHOS proteins, and proteins involved in the mitochondrial unfolded protein response, and increases in oxidative respiration, mitochondrial membrane potential, and ATP (Zhang et al., 2016). An alternative strategy for increasing SIRT1 activity is by administration of “sirtuin‐activating compounds” (STACs), that activate via an allosteric mechanism, and includes the naturally occurring polyphenol, resveratrol. One‐year‐old (middle‐aged) C57BL/6NIA mice fed a diet supplemented with resveratrol for 6 months showed increased survival up through 2.1 years of age, and increased performance on a rotarod (reflecting neuronal and/or muscle performance) [Baur et al., 2006]. However, feeding resveratrol to 18‐month‐old C57BL/6 mice for 10 months did not reduce sarcopenia: It did not reduce the age‐associated loss of muscle mass or improve maximal isometric force production; nonetheless, it did enhance mitochondrial SOD activity and reduce hydrogen peroxide and lipid peroxidation in muscle (Jackson, Ryan, & Alway, 2011). The synthetic STAC, SRT1720, given to C57BL6/J mice beginning at 6 months and continuing for the rest of their lives, improved mean lifespan by 8.8%, showed a modest increase in rotarod performance at 24 months, and by microarray analysis, changes in mRNA expression in muscle that could result in less inflammation (Mitchell et al., 2014). In summary, the jury is still out as to whether resveratrol or synthetic STACs are beneficial for treating sarcopenia in rodents.

## 3 DROSOPHILA

### 3.1 The model

Drosophila have been used as a model organism for aging research since 1913 when Roscoe Hyde observed and outlined how crossing two inbred lines led to extension of lifespan (Hyde, 1913). The low cost of maintenance, the absence of regulatory oversight for their use in experiments, the ease of generating large populations, its distinct tissues that can be dissected and genetically manipulated, and a large collection of readily available genetic tools, including CRISPR reagents for genome editing as well as constructs for overexpressing or knocking down any gene in a tissue and in a time‐specific manner, make Drosophila an excellent model for aging research (Kennedy & Partridge, 2017). In flies, muscles contribute a large percentage of body mass and they have many structural similarities with those of mammalian muscles. Additionally, muscle function is easily assayed by measuring their ability to fly and climb (Gargano, Martin, Bhandari, & Grotewiel, 2005). One major difference between Drosophila and mammalian muscle is the absence of muscle stem cells (Demontis, Piccirillo, Goldberg, & Perrimon, 2013). This feature makes Drosophila muscle, and as later described, *C*.* elegans* muscle, excellent models for identifying the mechanisms by which assembled sarcomeres are maintained and repaired without the confounding influence of regeneration as found in mammalian muscle.

### 3.2 Molecular discoveries

Like what has been found in rats, analysis of gene expression changes in *Drosophila* muscles during aging has shown increased expression of 26S proteasome components as well as increased expression of antioxidant stress response elements (Wheeler, Bieschke, & Tower, 1995). Another group found that the overexpression of FOXO (forkhead box O), an important transcription factor associated with longevity regulation (Murtaza et al., 2017), reduced the age‐related accumulation of p62–poly‐ubiquitin protein aggregates in fly muscles and preserved muscle function (Demontis & Perrimon, 2010). P62 is an autophagy receptor that also binds to poly‐ubiquitinated proteins, enabling ubiquitinated proteins to aggregate and connecting the ubiquitin–proteasome system to the autophagy–lysosome system (Liu et al., 2016). FOXO is the near‐terminal output of the insulin/insulin growth factor signaling pathway and is inhibited by Akt (protein kinase B) phosphorylation via this pathway (Sasako & Ueki, 2016). Antagonizing insulin signaling via transcriptional induction of the *Drosophila* ortholog of insulin‐like growth factor binding protein 7 caused lifespan extension and prevented age‐related muscle deterioration (Owusu‐Ansah et al., 2013).

An age‐related reduction in available calcium, which is necessary for muscle contraction, is a possible contributor to sarcopenia pathology. Lorenzo et al. found that in Drosophila, there is a decrease in sarcoplasmic reticulum calcium with age that correlates with the decline in muscle function. They hypothesized that this decline was due to increased leakiness of the ryanodine receptor calcium channel allowing more calcium to leave the sarcoplasmic reticulum (Delrio‐Lorenzo, Rojo‐Ruiz, Alonso, & Garcia‐Sancho, 2020).

### 3.3 Therapeutic discoveries

In line with what has been found in rats, Katewa el at. have shown that dietary restriction alleviates flight defects in aged flies (Katewa et al., 2012). The authors show that this is likely due to increasing mitochondrial function and fatty acid oxidation in the predominately aerobic flight muscles (Katewa et al., 2012). However, dietary restriction in flies does not prevent the senescence of the primarily glycolytic muscles used for walking and climbing (Bhandari, Jones, Martin, & Grotewiel, 2007). These and previous findings suggest that dietary restriction is useful for extending lifespan and preserving some, but not all, types of muscle cells. Sonowal et al. demonstrated positive effects of indole on motility (reflecting improved muscle and/or neuronal function) of elderly *Drosophila*. While they saw no difference in climbing motility in young flies, aged flies (20 days old) treated with indole or indole‐producing bacteria were approximately twofold more motile compared with age‐matched controls (Sonowal et al., 2017). They found that the benefits gained by indole treatment were dependent on the aryl hydrocarbon receptor (AHR), a conserved receptor for xenobiotic small molecules, and known to bind to indoles (Sonowal et al., 2017).

## 4 *Caenorhabditis elegans*

### 4.1 The model

*Caenorhabditis elegans* is an excellent genetic model organism to study sarcomere assembly, maintenance, and regulation (Gieseler, Qadota, & Benian, 2017), and conserved mechanisms of aging (Kenyon, 2010). The *C*.* elegans* model provides the shortest lifespan and largest possible sample size among the models described in this review. Their short lifespan (average: 18–21 days) makes them particularly convenient for aging studies. Muscle function is easy to monitor in worms since they require functioning body wall muscles for locomotion. The optical transparency of *C*.* elegans* can be exploited for mutant and drug screens (e.g., based on fluorescently tagged proteins expressed at endogenous levels via CRISPR). Additionally, like *Drosophila*, nematode muscle does not contain stem cells and thus provides an opportunity to investigate how the assembled muscle contractile apparatus is maintained and functions during aging in the absence of regeneration. Monica Driscoll's laboratory was the first to report that *C*.* elegans* undergo an age‐dependent decline in whole animal locomotion and deterioration of the muscle myofilament lattice, and thus, *C*.* elegans* is a good model for sarcopenia (Herndon et al., 2002). They found variability among same‐age animals, even of advanced age, in the time of onset and severity of reduced locomotion. Because these animals have the same genomes (i.e., they are isogenic), this indicated that like humans, a major factor in nematode aging is stochastic.

### 4.2 Molecular discoveries

Through genetic analysis, it has been discovered that many genes that when mutated result in lifespan extension in *C*.* elegans* (Kenyon, Chang, Gensch, Rudner, & Tabtiang, 1993; Kenyon, 2010). A major pathway that affects longevity is the insulin receptor pathway: Loss‐of‐function mutations in the single insulin receptor, DAF‐2, or in the downstream PI(3)kinase, AGE‐1, result in lifespans that are 2‐2.5X longer, respectively, compared with wild‐type animals. Herndon et al. reported that in an *age*‐*1* mutant, there was significant delay in the onset of the dysmorphology of muscle nuclei compared with wild type (Herndon et al., 2002). This same *age*‐*1* mutant also delays loss of locomotion in aging worms (Duhon & Johnson, 1995). A DAF‐2 (insulin receptor) mutant was found to have a transcription‐dependent increase in muscle mass and abundance of key sarcomere proteins, such as myosin (Depuydt et al., 2013). Loss of function of *daf*‐*2* results in an induction of mitophagy in body wall muscle, including an increase in level of the mRNA for DCT‐1, the nematode ortholog for mammalian BNIP3, and the mitophagy receptor (Palikaras, Lionaki, & Tavernarakis, 2015). Thus, induction of mitophagy, a quality control mechanism for mitochondria, might partly account for the preserved muscle function in older *daf*‐*2* mutant animals.

*Caenorhabditis elegans* microarray analysis has revealed 27 genes encoding evolutionarily conserved muscle sarcomere proteins that undergo a twofold decrease in mRNA expression between day 0 and day 7 adults (Mergoud Dit Lamarche et al., 2018). This decline occurs as early as day 1 of adulthood, which is perhaps not surprising since sarcomere assembly is completed by day 0 of adulthood (Gieseler et al., 2017) and sarcomeres are quite stable structures (Solomon & Goldberg, 1996). To understand the mechanism by which there is an early decline in sarcomeric protein transcripts, Lamarche et al. examined the transcript levels during aging of two myogenic transcription factors, UNC‐120 (serum response factor/SRF) and HLH‐1 (MyoD) that are known to be required for embryonic muscle differentiation, but are also expressed in adult muscle. They found that between day 0 adults and day 7 adults, there was a 40% decrease in the level of *unc*‐*120* mRNA, but a weak increase in the level of *hlh*‐*1* mRNA. They found that changes in *unc*‐*120* expression did not affect lifespan; however, reducing *unc*‐*120* expression via RNAi accelerated muscle aging, and conversely, overexpression of *unc*‐*120* delayed muscle aging (Mergoud Dit Lamarche et al., 2018). Interestingly, markers of muscle aging included an age‐dependent increase in mitochondrial fragmentation (usually associated with decreased function, see below) and an increase in autophagic vesicles. Lamarche et al. also saw a two‐ to eightfold increase in sarcomere transcripts at day 5 of adulthood in a *daf*‐*2* mutant. This mutant also showed a twofold increase in *unc*‐*120* transcripts from day 1 through day 5. They found via RNAi of *unc*‐*120* that the beneficial effects of the *daf*‐*2* mutation on muscle aging are dependent on *unc*‐*120*. This suggests that the downstream DAF‐16 (FOXO) transcription factor may regulate the *unc*‐*120* promoter.

Several studies have shown that mitochondrial organization and function decline in body wall muscle of nematodes as they age. Yasuda et al. showed that by EM, there is a progressive enlargement and swelling of mitochondria in body wall muscle (Yasuda et al., 2006), similar to what has been shown in mouse muscle (Leduc‐Gaudet et al., 2015). In a carefully performed study, Gaffney et al. showed that by assessing mitochondrial network structure and A‐band organization using fluorescence microscopy, there is a progressive loss of mitochondrial and A‐band organization from adult day 0 through adult day 16 (Gaffney et al., 2018). Interestingly, the authors show that mitochondrial fragmentation is evident at day 4, whereas A‐band disorganization is evident at day 6 and that the extent of the mitochondrial defect is better correlated with the decline in whole animal locomotion than is the extent of the A‐band defect. Intriguingly, the authors demonstrated that a loss of mitochondrial function precedes and thus may result in loss of mitochondrial structure: They observed a decline in “maximal mitochondrial ATP production rate” from day 0 to day 2 of adulthood, and a loss of mitochondrial membrane potential from day 0 to day 4 of adulthood. One weakness of their study, however, is that they performed these mitochondrial function experiments on mitochondria purified from whole nematodes. It will be important to determine whether the same results can be obtained using mitochondria isolated from body wall muscle, using a recently developed method (Ahier et al., 2018). Nevertheless, this leads to an intriguing possibility that the decline in mitochondrial function may explain the onset and progression of loss of muscle function before there is a decline in sarcomere organization.

### 4.3 Therapeutic discoveries

Like rodents, flies, and most other species (Masoro, 2005), dietary restriction leads to increased longevity of *C*.* elegans* through multiple pathways (Greer & Brunet, 2009). Depuydt et al. found that dietary restriction causes an increase in muscle mass and abundance of integral sarcomere proteins, like the different myosin isoforms (Depuydt et al., 2013). They found that this increase in sarcomere proteins is transcription‐independent and theorizes that it is caused by selective inhibition of structural muscle protein degradation or an increase in muscle‐specific protein synthesis. Like in mammals, exercise has been shown to have a modest, yet significant, positive effect on the lifespan and muscle function of *C*.* elegans* (Chuang, Kuo, Lee, Chu, & Chen, 2016; Hartman et al., 2018). The report from Hartman et al. is particularly intriguing. Starting from adult day 2, they forced the worms to undertake swimming as exercise twice a day (90 min each) for 6 consecutive days and then assessed mitochondrial health on day 12. Exercise clearly led to less fragmentation of body wall muscle mitochondria, and less whole animal lethality from the “mitotoxicants” rotenone and arsenic, as compared to animals that had not exercised, but it did not increase the mitochondrial DNA copy number, or reduce the extent of mitochondrial DNA lesions, or increase basal respiration.

The naturally occurring compounds urolithin A and indole—discussed above—also improve the muscle function in aged *C*.* elegans* (Ryu et al., 2016; Sonowal et al., 2017). Urolithin A extended the worms' lifespan and prolonged normal activity—crawling mobility and pharyngeal pumping—via inducing mitophagy and improving the mitochondrial health of body wall muscle mitochondria (Ryu et al., 2016). Sonowal et al. found that treating with indole or indole‐producing bacteria also improved whole animal locomotion and pharyngeal pumping of older adults (presumably due to improved muscle function) but did not increase the lifespan of *C*.* elegans*. They found that, like in Drosophila, the effects of indole are dependent on the aryl hydrocarbon receptor, AHR‐1 (Sonowal et al., 2017). However, it was not determined whether the improved mobility was due to improvements in muscle and/or neuronal function.

## 5 ZEBRAFISH

### 5.1 The model

While an established tool for studying myopathies and muscular dystrophies, zebrafish are a relatively newer model of aging and sarcopenia compared with the other models discussed in this review. Zebrafish were presented as an aging model in 2002 by Gerhard et al. who investigated the process of zebrafish aging and later demonstrated that it is comparable to human aging and suitable to study age‐dependent changes in musculoskeletal function (Gerhard, 2003; Gerhard & Cheng, 2002; Gerhard et al., 2002). Zebrafish as a model for aging has also been supported by the work of Kishi et al. (Kishi, 2004; Kishi et al., 2003) The ease of care, prolific breeding, easy genetic manipulability, and ability to readily absorb drugs through their water make the zebrafish an attractive model to work with (Daya, Donaka, & Karasik, 2020; Maves, 2014; Volpatti et al., 2020). Additionally, zebrafish skeletal muscle makes up a large portion of the fish trunk and has a high degree of similarity with human muscle, including the presence of satellite‐like cells for muscle repair and regeneration (Gurevich et al., 2016; Hollway et al., 2007). Zebrafish do have a longer lifespan (average of 42 months) compared with other models discussed in this review, which is a downside of using this model for aging studies (Gerhard et al., 2002). While the zebrafish model has not been utilized in sarcopenia studies as much as other models, it is a very promising vertebrate model for studying muscle aging going forward.

### 5.2 Discoveries

There have been few discoveries made concerning aged muscle using zebrafish. Gilbert et al. have shown that, like in humans, exercise training improves swimming performance in young (8‐12 months) and middle‐aged (15–20 months) fish, but not old fish (25–30 months)—providing further evidence that exercise alone is not sufficient to treat sarcopenia (Gilbert, Zerulla, & Tierney, 2014).

## 6 DISCUSSION

In this review, we have discussed the benefits of rodent, fly, nematode, and zebrafish models in the context of sarcopenia research (summarized in Figure 1). *Drosophila* and *C*.* elegans* are both inexpensive, short‐lived models that more easily provide molecular and genetic insights. Rats and mice are more expensive and time‐consuming, but more closely model human muscle and are better suited for testing therapeutic strategies. Zebrafish are inexpensive, yet longer‐lived and are a relatively new model to consider for sarcopenia studies.

There are multiple conserved cellular pathways and mechanisms that appear to be involved in sarcopenia pathogenesis across multiple model organisms. In *C*.* elegans*, *Drosophila*, and mice, there is evidence that the insulin/IGF1 and Akt signaling pathways appear to play a role in both aging and the age‐related decline in muscle health through FOXO transcription factor(s). Data from these three models provide evidence for identifying compounds that increase FOXO nuclear translocation as a possible therapeutic for sarcopenia patients. The quality of the skeletal muscle mitochondria is another conserved factor to consider when searching for sarcopenia therapeutics. Mitochondria quality is reduced in both *C*.* elegans* and rodent aged muscle, and there is evidence in *C*.* elegans* that this precedes the decline in muscle function with age. Additionally, the loss of muscle mass may, at least in part, be explained by the increase in protein degradation via the proteasome system. In both Drosophila and rats, components of the 26S proteasome were elevated in aged animals.

Currently, the NIH clinical trials database (clinicaltrails.gov) shows over 400 studies related to sarcopenia with 23 active and 177 completed interventional studies. Sarcopenia research would greatly benefit from the use of *Drosophila* and *C*.* elegans* to identify more therapeutic strategies and the use of rodents to then test those therapies further. Such an “evolutionary pipeline” approach was reported recently to identify small molecules that might be useful for treating RYR1‐related myopathies (Benian & Choo, 2020; Volpatti et al., 2020). Currently, research in rodents has been focused on muscle regeneration instead of muscle maintenance despite the known depletion of muscle satellite cells during aging (Garcia‐Prat, Sousa‐Victor, & Munoz‐Canoves, 2013; Jones & Rando, 2011). Without the normal population of muscle satellite cells or a way to replenish the satellite cells with age, research may need to shift focus to how existing muscle cells (“fibers”) can be better maintained. Both *Drosophila* and *C*.* elegans* are well‐suited models for muscle maintenance since they do not have any muscle satellite cells. High‐throughput screenings to identify drugs, small molecules, or conditions that improve muscle maintenance with aging can be done easily with both flies and worms. Candidate therapies for sarcopenia can be identified in this way. Any treatments that alleviate muscle aging in these models will be independent of regeneration through satellite cells and, thus, possibly more suitable for aged individuals who have lost much of their satellite cell population. These types of treatments may also be beneficial for the maintenance of cardiac muscle, which does not possess stem cells and the ability to regenerate.

## CONFLICT OF INTEREST

The authors declare that they have no conflicts of interest.

## AUTHOR CONTRIBUTIONS

The initial idea for this review was from G.M.B. C.J.C. did the majority of the literature searches and created the initial draft, including its organization. Then, G.M.B. wrote some sections, and each author edited what the other had written, and this process of editing went through multiple iterations, including additions made in response to the reviewer's suggestions. Both authors approved the final version of the manuscript.

## Data Availability

Data sharing is not applicable to this article as no new data were generated or analyzed.
